# The relationship between smoking cessation and vitamin D

**DOI:** 10.1007/s11845-026-04290-6

**Published:** 2026-02-28

**Authors:** Meryem Betos Koçak, Duygu Kavuncuoğlu

**Affiliations:** 1 Department of Family Medicine, Health Sciences University, Faculty of Medicine Balıkesir Atatürk City Hospital, Balıkesir, Turkey; 2 Balıkesir Provincial Health Directorate Balıkesir, Balıkesir, Turkey

**Keywords:** Smookig cessation, Vitamin D level, Smooking cessation outpatients clinic

## Abstract

**Background:**

Smoking addiction is the most important reversible risk factor affecting many organs, including the heart, lungs, and central nervous system. Smoking cessation alone should not be the sole focus of smoking addiction, and comorbidities should be considered.

**Aims:**

In our study, the relationship between successful smoking cessation and vitamin D levels was analyzed.

**Methods:**

This was a retrospective cohort study. Our study included patients who visited the smoking cessation outpatient clinic. All patients were started on the same dosage of cytisine active ingredient at the same time. After one month of treatment, smoking cessation success and vitamin D levels were compared. In addition, demographic data, the amount and duration of smoking, and smoking cessation success after treatment were recorded.

**Results:**

Our study included 327 patients and the smoking cessation rate after treatment was 58.7%. A statistically significant correlation was found between vitamin D level and smoking cessation success. We found that patients with higher vitamin D levels had a higher smoking cessation success rate

**Conclusion:**

Many studies have reported a relationship between addiction and vitamin D level. Vitamin D supplementation has been used in many addiction treatments, and positive results have been obtained with this treatment. In our study, we found that patients with low vitamin D levels had low smoking cessation success rates. In our study, we found a relationship between high vitamin D levels and smoking cessation success and suggested that vitamin D supplementation should be initiated in patients with low vitamin D levels to increase the smoking cessation rate.

## Introduction

Smoking is the most important preventable cause of morbidity and mortality worldwide and causes eight million deaths annually. Smoking causes mortality by affecting many systems and morbidity by impairing the mental status [1]. From the moment of smoking, acute nicotine and carbon monoxide exposure increases heart rate and blood pressure, vasoconstricts, decreases oxygen-carrying capacity, narrows airways, and increases cough and irritation [2, 3]. Smoking is an important risk factor for chronic diseases(e.g., malignant tumors, cardiovascular diseases respiratory diseases, tuberculosis, and stroke [4].

In 2012, smoking-related diseases were responsible for an estimated purchasing power parity(PPP) $467 billion in healthcare spending, representing about 5.7% of global health expenditures. When both medical costs and productivity losses were combined, the overall economic burden of smoking reached PPP $1852 billion, corresponding to approximately 1.8% of the world’s annual gross domestic product. Smoking also imposes a huge economic burden on the society [5]. The global economic costs associated with tobacco use are estimated to exceed USD 1 trillion annually. The treatment costs for patients who smoke are estimated to be USD 10,700 more per patient than those for patients who do not smoke. In the United States, approximately $ 300 billion is spent annually on smoking-related diseases [6, 7]. A comprehensive economic analysis examining the direct cost burden of smokers on the healthcare system reported that healthcare costs for smokers are approximately 40% higher than for nonsmokers in the same age group [8]. Smoking increases the risk of hospitalization and the length of hospital stay [9]. Despite the many negative effects of smoking, smoking-related damage can be reversed in some individuals by stopping smoking. For this reason, smoking cessation is promoted as a key strategy to halt disease evolution before irreversible changes ocur [1, 4]. In addition, positive effects of smoking cessation on health have been observed [10]. After quitting smoking, improvement in lung function, improvement in cardiovascular function, elimination of constipation, reduction in mouth ulcers and short-term weight gain, as well as improvement in the activity of cytochrome P450 enzyme are observed [11].

In addition to behavioral support, pharmacological treatment is a key strategy for increasing the effectiveness of smoking cessation interventions. Varenicline, nicotine replacement therapy (NRT), and bupropion are recommended as first-line treatment options. Combination NRT, which uses a patch with a short-acting nicotine form, is also an effective alternative. Pharmacotherapy, when combined with behavioral interventions, maximizes smoking cessation success. WHO clinical treatment guideline for tobacco cessation in adults [12]. Two treatment methods, psycho-behavioral and pharmacotherapy, are used for smoking cessation [10]. Research on which of these two methods is more effective is ongoing. Smoking cessation treatments differ in their effects on smoking cessation due to different content, intensity, approach, and application methods [4]. The most effective way for patients to quit smoking is currently unclear [13]. The article, which includes 363 studies for efficacy and 355 for safety, emphasizes the difficulties in determining a single definitive option for the “best treatment os smoking cessation” [14]. However, smoking addiction treatment should not focus solely on smoking cessation. Other factors may affect smoking cessation and are currently being evaluated by doctors, healthcare professionals. Other factors that may affect smoking cessation should also be evaluated. Mental disorders, such as anxiety, depression, and schizophrenia, have also been observed in many smokers [15]. In these patients, treatment of diseases such as depression and schizophrenia should be carried out simultaneously. Cigarette use is highly prevalent among individuals participating in substance abuse treatment programs, and smokers are reported to have higher psychiatric symptoms, impulsivity, and substance use severity [16].

It has been reported that the rate of smoking among substance addicts is between 74 and 98% [17]. Smoking cessation treatment should not be considered alone for substance addicts; substance addiction treatment should be applied to these patients in addition to smoking cessation treatment [15]. It is emphasized that cigarette smoking is highly comorbid with substance use disorders, which can complicate treatment. It has also been shown that there is a significant association between nicotine product use and alcohol and other drug use disorders [18, 19]. Vitamin D has many functions in the body, and new functions are being identified [20]. Vitamin D deficiency has been associated with many diseases (central nervous system, cardiovascular, musculoskeletal, and systemic inflammatory diseases) [21].

Considering that patient-specific factors are important for smoking cessation success, we aimed to investigate the effect of vitamin D deficiency on smoking cessation success. To the best of our knowledge, our study is the first to investigate the relationship between successful smoking cessation and vitamin D.

## Material and method

### Study design and participants

This was a retrospective cohort study. Our study was conducted on patients were admitted(outpatient treatment) to the Smoking Cessation Outpatient Clinic of Balıkesir Atatürk City Hospital. People who applied between 01/04/2024-30/11/2024 were included in our study.

The patients included in the study were classified according to age, gender, cardiovascular disease (ischemic heart disease, heart failure, arrhythmia) and lung disease (COPD, pulmonary fibrosis, bronchiectasis, atelectasis), and smoking was classified as less than or more than 20 cigarettes per day. The duration of smoking was classified as under and over 20 years. Vital signs (blood pressure, pulse rate, temperature, respiratory rate, oxygen saturation), smoking cessation status, and vitamin D (25-hydroxy vitamin D, 25(OH)D) level at the time of presentation to the smoking cessation outpatient clinic were recorded. Because of its structural stability and long half-life in circulation, serum or plasma concentrations of 25(OH)D are preferred indicators of vitamin D status [22, 23]. In our study, we used 25(OH)D level as an indicator of vitamin D. Vitamin D levels were recorded as above or below 20 ng/mL based on the generally accepted lower limit value of 20 ng/mL [24, 25]. All these data were obtained from the patient files created separately for each patient during the first assessment at the smoking cessation outpatient clinic and from the hospital automation system. The inclusion and exclusion criteria were shown in the Table 1.

**Table 1:** The inclusion and exclusion criteria

INCLUSION CRITERIA	EXCLUSION CRITERIA
Ages of 18 to 65 years	Patients for whom medication is not considered appropriate by physicians.
Visit a smoking cessation clinic	Patients refusing medication
	Patients who did not regularly use smoking cessation medication started at the Smoking Cessation Outpatient Clinic or who stopped using the medication
	Patients who do not come to patient check-ups
	Missing or incorrect data in patient files
	Pregnancy and breastfeeding
	Patients taking vitamin D medication during the study
	Patients in whom vitamin D levels could not be available
	Patients taking drugs that affect vitamin D metabolism (phenobarbital, carbamazepine, dexamethasone, nifedipine, spironolactone, clotrimazole, and rifampin)
	Chronic liver, kidney, and parathyroid gland disease
	Diseases causing gastrointestinal malabsorption (short bowel syndrome, pancreatitis, inflammatory bowel disease, amyloidosis, celiac disease, and malabsorptive bariatric surgery)
	Osteoporosis

### Procedure

Data were obtained from patient files and the hospital’s automation system. Patient files were filled by a physician for each patient who applied to the smoking cessation outpatient clinic. The physician completed them during an in-clinic evaluation. The smoking cessation outpatient clinic is staffed by physicians authorized by the Ministry of Health of the Republic of Turkey. Patients who visit the smoking cessation outpatient clinic are treated by certified physicians. Medical treatment is determined by the Ministry of Health of the Republic of Turkey and is provided free of charge to patients deemed appropriate by physicians. Treatment is given by physicians who examine patients. During our study, medication with the active ingredient, cytisine, was used for medical treatment. Cytisine was administered to all patients using the same procedure. All patients were treated for the same duration (1 month) at the same dosage. The smoking cessation status of all patients was recorded at the end of 1 month.

Approval was obtained from the local ethics committee for the study. It was written in accordance with the Declaration of Helsinki (decision number: 2024/09/48, date: 19/09/2024).

### Statistical analysis

Statistical analyses were performed using IBM SPSS Statistics for Windows version 23 statistical analysis program (IBM). Data are presented as the mean, standard deviation, median, minimum, maximum, percentage, and number. The normal distribution of continuous variables was evaluated using the Shapiro-Wilk and Kolmogorov-Smirnov tests. An independent sample t-test was used to compare normally distributed data between two independent groups, and the Mann-Whitney U test was used to compare non-normally distributed data. Categorical variables were compared using the chi-square and Fisher’s exact tests. Statistical significance was set at p value < 0.05.

## Result

**Fig. 1:**
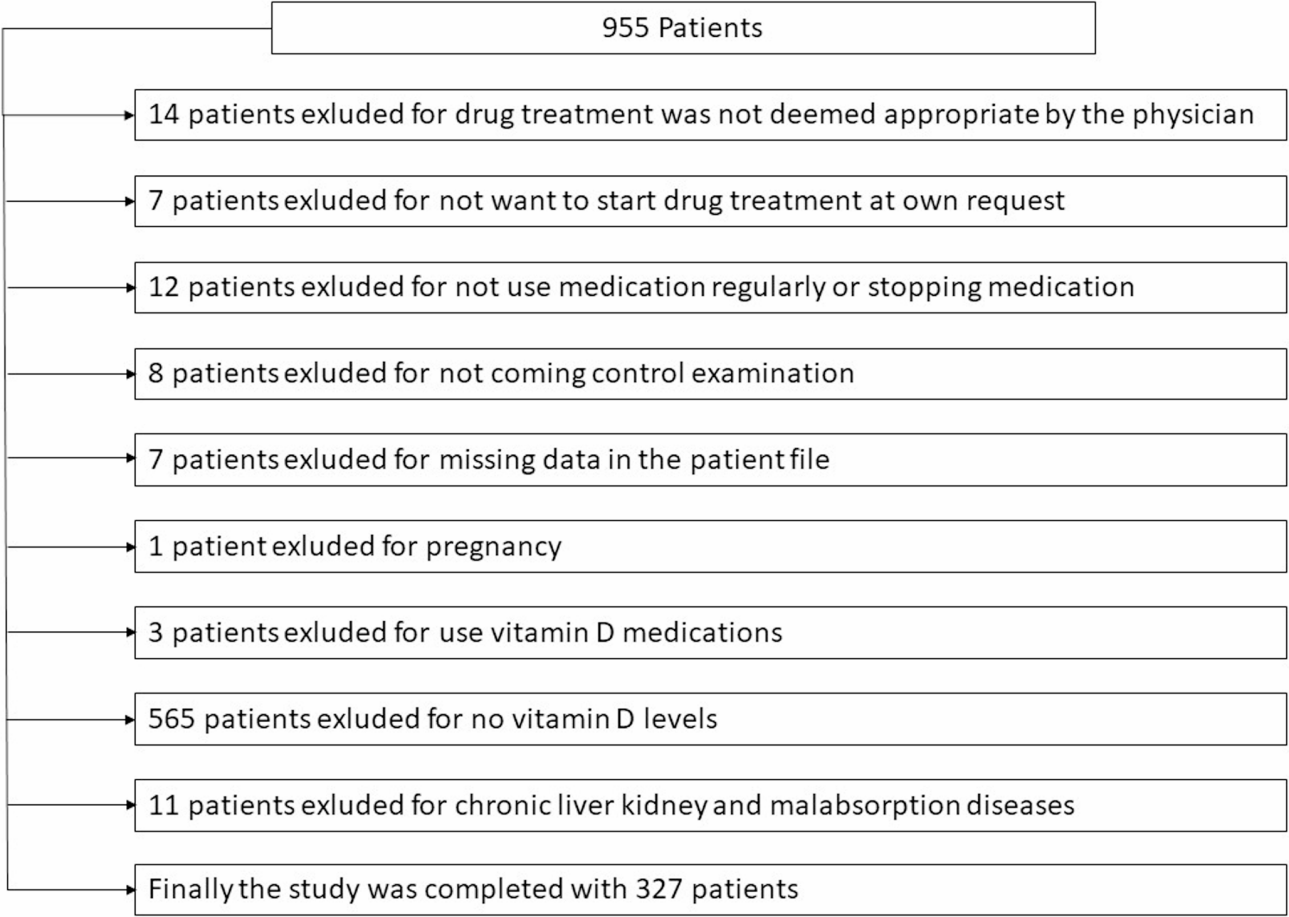
A flowchart of the study

Our study included patients who visited the smoking cessation outpatient clinic within an 8-month period. While 955 patients applied to the smoking cessation outpatient clinic during this period, the study was completed with 327 patients when the exclusion criteria were applied. A flowchart of the study is shown in Fig. 1.

The patients included in our study, with a mean age of 43.99 (± 12.64). Of the patients included in the study, 209 (63.9%) were male. The study participants presented with the following comorbidities: cardiovascular disease in 211 patients (64.5%) and pulmonary disease in 114 patients (34.9%). The number of cigarettes smoked per day was as follows: 105 (32.1%) patients smoked 1–20 cigarettes/day and 222 (67.9%) patients smoked 21 or more cigarettes per day. The number of patients who smoked for 1–20 years was 180 (55%) and the number) had smoked for > 20 years. The patients’ vital signs are shown in Table 2.

**Table 2:** The vital signs of the patients

	Minimum	Maximum	Mean	Standard Deviation
Systolic Blood Pressure (mmHg)	110	157	134,22	13,11
Diastolic Blood Pressure (mmHg)	54	100	76,62	7,887
Pulse (beats/min)	60	122	88,56	8,189
Oxygen Saturation(%)	81	99	92	4,855
Fever(^0^C)	35,8	36,9	36,404	,2326
Respiratory Rate(min)	10	23	15,60	2,580

Of the 327 patients included in the study, 192 (58.7%) had quit smoking at the end of treatment. May 2024, 26 patients, June 2024, 29 patients, July 2024, 28 patients, August 2024, 26 patients, September 2024, 27 patients, October 2024, 29 patients, November 2024, 27 patients quit smoking.

Regarding the patients included in the study, there was no statistically significant relationship between age, sex, and smoking cessation success (*p* > 0.05). There was no statistical relationship between the vital signs (systolic and diastolic blood pressure, pulse rate, oxygen saturation, temperature, and respiratory rate) and smoking cessation success (*p* > 0.05). There was also no statistical relationship between comorbidities (CVS, pulmonary) and smoking cessation in the 327 patients (*p* > 0.05). In our study, there was no relationship between the number of cigarettes smoked per day and successful smoking cessation (*p* > 0.05). In our study, a statistically significant relationship was detected between smoking for 20 years or less and smoking cessation success (*p* < 0.05).

The average vitamin D level of the 317 patients included in the study was 17.38 ng/mL ± 9.72 (minimum value 3.1, maximum value 67.6 ng/mL). The average vitamin D level of 110 patients (34,7%) with a vitamin D level of 20 or higher was 27.71 ng/mL ± 9.06. The extreme values ​​of these patients ranged from 20.20 to 67.60 ng/mL. The average vitamin D level of 217 patients (65,3%) with a vitamin D level of less than 20 was 12.14 ng/mL ± 4.40. The extreme values ​​of these patients ranged from 3.10 to 19.70 ng/mL.

In the evaluation of vitamin D levels, 110 patients had vitamin D levels ≥ 20 ng/mL. 78 of these patients (70,9%) achieved smoking cessation after treatment. Thirty-two patients with vitamin D levels of ≥ 20 ng/mL did not quit smoking. A statistically significant correlation was found between smoking cessation success in patients with vitamin D levels ≥ 20 ng/mL (*p* < 0.05).

## Discussion

Vitamin D is a fat-soluble sterol [26]. Vitamin D is transported to target tissues by vitamin D binding protein (DBP). The primary function of vitamin D is to regulate phospho-calcium metabolism to maintain normal calcium levels and to protect bone development. However, vitamin D receptors (Vitamin D Receptor, VDR) are found in many tissues other than the musculoskeletal system, such as the prostate, brain, breast, pancreas, and immune cells. Vitamin D plays a role in the regulation of immune functions, cell proliferation or cell differentiation in these tissues. Vitamin D may influence the risk of many cardiometabolic diseases including hypertension, cardiovascular disease, and diabetes mellitus [27]. An association between low vitamin D levels and bacterial pneumonia has been previously reported [28]. Vitamin D supplementation reduces the risk of autoimmune diseases, particularly rheumatoid arthritis and polymyalgia rheumatica [25]. Vitamin D is an important immunomodulator, and its association with autoimmune and inflammatory diseases, such as multiple sclerosis, has also been demonstrated [29]. There is a relationship between vitamin D levels and several diseases.

A study by Musazadeh et al. suggests that low vitamin D levels may increase the risk of depression, while vitamin D supplements may reduce symptoms of depression [30]. In another article, vitamin D supplementation was shown to reduce depressive symptoms compared to placebo in people with depression [31]. A meta-analysis including observational and epidemiological studies reported a positive association between low vitamin D and the risk of depression [32]. Many authors have also suggested a relationship between psychiatric and low vitamin D levels. Oxidative stress and inflammation have been reported to play roles in the pathophysiology of mood disorders [33–35]. According to the results obtained from a systematic review by Adamson et al., patients with psychotic disorders had significantly lower vitamin D levels than the healthy control group [36]. Vitamin D is a positive immunomodulator, and because of this, benefits may be obtained from its use in mood disorders [33]. In a 2022 meta-analysis of randomized controlled trials, vitamin D was shown to have beneficial effects on both the incidence and prognosis of depression [37]. In a meta-analysis of 25 studies (*n* = 7534) evaluating the effectiveness of vitamin D supplementation in depression, vitamin D was reported to be effective in individuals with major depressive disorder [35]. In a randomized controlled clinical trial evaluating the effectiveness of vitamin D supplementation on anxiety, vitamin D administration improved anxiety symptoms [38].

Since there is no article in the literature examining the relationship between smoking cessation success and vitamin D, we examined smoking addiction through the relationship between substance addiction and vitamin D. A relationship between the effects of vitamin D deficiency on the central nervous system and opioid addiction has been reported [39]. There is no clear consensus on the mechanism of the relationship between substance addiction and vitamin D; however, the most widely accepted view is the effect of vitamin D on dopamine release in the central nervous system. Dopaminergic neurons are associated with many diseases (depression, psychosis, mania, Parkinson’s disease, Tourette syndrome, obsessive compulsive disorder, attention deficit hyperactivity disorder, anxiety, hallucinations, and delusions). Dopaminergic drugs are also used to treat these disorders. In one study, an increase in dopamine transporter protein expression was found in addiction [40]. This demonstrates a relationship between addiction and dopamine levels [40]. Vitamin D protects dopaminergic neurons against neurotoxic effects [41].

In this study, vitamin D administration was shown to reduce dopaminergic neuron loss, oxidative stress, and inflammation; preserve dopamine content; and reverse behavioral changes [42]. Another study showed that vitamin D has a regulatory effect on the development, differentiation, and survival of dopaminergic neurons; it increases dopamine production and release, improves neuronal morphology, and supports synaptic functions [43]. Dopamine-producing cells in the brain are affected by vitamin D. Vitamin D deficiency can impair the differentiation of dopamine neurons and the expression of enzymes in the dopamine production pathway [44]. It has been suggested that vitamin D deficiency may be associated with many neurological and neurodegenerative diseases—particularly through the dopaminergic system, neuroinflammation, and neurodegenerative processes [44, 45]. It has been reported in the literature that low vitamin D levels cause a decrease in dopamine in the central nervous system. Dopamine depletion is also known to be associated with many central nervous system disorders. Our study also found that those with low vitamin D levels had lower smoking cessation rates than those with normal vitamin D levels. This may be due to the effect of vitamin D levels on dopamine expression.

In an animal study conducted by Eserian in 2013, newborn female rats were administered a single dose of vitamin D, and it was found that the amount of dopamine and its metabolites increased in the brain stem, hypothalamus, and striatum of these rats in adulthood. This suggests that vitamin D increases the dopaminergic activity in the brain [46, 47]. In addition, Vitamin D is effective in the synthesis of some neurotransmitters (serotonin, dopamine, noradrenaline, and adrenaline) by activating tyrosine hydroxylase, which is reported to be the rate-limiting step in catecholamine synthesis [33]. A decrease in vitamin D levels leads to a decrease in the levels of these neurotransmitters [33]. In our study, we found that patients with low vitamin D levels had lower smoking cessation success. We believe that higher smoking cessation success can be achieved by achieving a dopaminergic effect by starting vitamin D support.

Vitamin D, also called neurosteroid hormone due to its immunomodulatory and neurotrophic roles [33]. Studies has been reported to affect behavior in humans by regulating the release of neurotransmitters and the synthesis of neurotrophic factors [48, 49]. In addition, vitamin D has recently been found to have neuroprotective effects against cognitive decline [50]. Considering the effects of vitamin D on human behavior and cognition, There may be a possible association between vitamin D and substance use disorders [39, 51]. As smoking is also an addiction, there is a relationship between smoking and vitamin D levels. Therefore, starting additional vitamin D supplementation, especially in patients with low vitamin D levels, may positively affect smoking cessation.

Galyuk [40], presents two different patients in his article. Among these patients, the patient codenamed A, in a patient with severe cocaine use disorder and moderate alcohol use disorder, all depressive complaints disappeared with vitamin D supplementation without any special antidepressant medication. In the same article presented by Galyuk [40], the other patient, coded D, was reported to have stopped using methylphenidate and started to reduce/stop the antidepressant dose after using newly started vitamin D supplementation. In our study, people with normal vitamin D levels quit smoking with a higher success rate.

## Conclusion

Vitamin D is an exciting and rapidly developing area of ​​research. Vitamin D can be recommended as an adjunct therapy in the treatment of various diseases, particularly mental health/addiction. Vitamin D can be used as a single treatment. In our study, we found an association between low vitamin D levels and smoking. We suggest that initiating vitamin D supplementation in patients with low vitamin D levels may have a positive impact on smoking cessation success.

## Data Availability

The data supporting the fndings of this study are available from the corresponding author upon reasonable request.
